# [Corrigendum] TBL1XR1 as a potential therapeutic target that promotes epithelial-mesenchymal transition in lung squamous cell carcinoma

**DOI:** 10.3892/etm.2026.13247

**Published:** 2026-07-23

**Authors:** Yuehua Zhao, Hao Lin, Jingwei Jiang, Mengxi Ge, Xiaohua Liang

Exp Ther Med 17:91–98, 2019; DOI: 10.3892/etm.2018.6955

Following the publication of the above paper, an interested reader has drawn to the Editor’s attention a number of inconsistences reported in the text of the article. First, the Abstract appeared to contradict with what was reported in the Results text and Fig. 5 on p. 96, in the sense that, where the Abstract stated that: “Overexpression of TBL1XR1 significantly increased E-cadherin protein expression, whilst snail family transcriptional repressor 1 (SNAI1), zinc finger E-box binding homebox 1 (ZEB1), p-Smad2/3, Smad2 and Smad3 protein expression were significantly reduced, compared with the control group.”, the opposite regulatory trends were reported in the Results section and in Fig. 5. Secondly, the description of the experiments in the legend for Fig. 3D did not correlate with what was shown in the figure; moreover, the text featured here appeared to have been duplicated from the text featured in the legend for Fig. 3B. Thirdly, the antibody sources/catalogue information appeared to be internally inconsistent in the Materials and methods section; for example, several antibodies with catalogue numbers in the “ab*xxxx*” format (a numbering convention specific to Abcam) were simultaneously attributed to other suppliers. Fourthy (and finally), an inconsistent description of error bars (SEM vs. SD) was noted in the legends of Figs. 1, 2, 3, and 5.

The authors have revisited the text of their paper, and recognize that a number of errors went uncorrected before the paper was published. First, the Abstract reported the abovementioned results incorrectly (where the description of the findings in the Results section, and the data in Fig. 5 itself, were correct). The affected text in the Abstract should have read as follows: “Overexpression of TBL1XR1 significantly decreased E-cadherin protein expression, whilst snail family transcriptional repressor 1 (SNAI1), zinc finger E-box binding homebox 1 (ZEB1), p-Smad2/3, Smad2 and Smad3 protein expression were significantly increased, compared with the control group.” Note that this correction is limited to the wording in the Abstract, and does not affect any experimental data, data interpretation or the overall conclusions of the study.

Secondly, the text describing the experiments for Fig. 3D in the legend for Fig. 3 was inadvertently duplicated from the text for Fig. 3B during manuscript preparation (the experimental data and figure itself are correct, as shown). The text for Fig. 3D in the Fig. 3 legend should have read as follows: “(D) Effects of TBL1XR1 knockdown on H1703 cell invasion were determined using the Transwell invasion assay with chambers precoated with Matrigel^®^ (magnification, x200).”

Thirdly, the original manuscript contained inaccuracies in the reporting of the catalogue numbers, and their formatting. The Materials and methods section has now been thoroughly corrected to ensure that each antibody is accurately matched with its corresponding supplier and catalogue number, and the antibody information has been standardized in the revised manuscript. Note that these corrections only involve the documentation of reagents, and do not affect the experimental procedures, data or conclusions presented in this study. The details for the antibodies should have been written in the Materials and methods section as follows: “Anti-GAPDH (cat. no. sc-32233; Santa Cruz Biotechnology, Inc., Dallas, TX, USA), anti-snail family transcriptional repressor 1 (SNAI1; cat. no. 3879), anti-zinc finger E-box binding homeobox 1 (ZEB1; cat. no. 3396), anti-E-cadherin (cat. no. 3195), anti-mothers against decapentaplegic homolog 2 (Smad2; cat. no. 5339), anti-Smad3 (cat. no. 9523), and anti-phosphorylated (p)-Smad2/3 (cat. no. 8828; all Cell Signaling Technology, Inc., Danvers, MA, USA).”

Finally, the description of the error bars was accurately observed to have been inconsistent, comparing between the “*Statistical Analysis*” section and the figure legends. The error bars shown in all figures represented standard deviation; accordingly, the “*Statistical Analysis*”section has been revised to state that all quantitative data are presented as the mean ± standard deviation, ensuring consistency throughout the manuscript. The corrected “*Statistical Analysis*” section (on p. 93, left-hand column) should therefore have read as follows: “...all data [were] expressed as the mean ± standard deviation.”

The authors are grateful to the Editor of *Experimental and Therapeutic Medicine* for allowing them this opportunity to publish a Corrigendum, and all the authors agree with its publication. The authors also thank the reader for drawing these anomalies and inconsistencies to their attention, and apologize to the readership for any inconvenience caused.

